# Comparison of the level of physical activity after the COVID-19 pandemic in Poland, Slovakia and the Czech Republic

**DOI:** 10.1186/s13102-024-00833-5

**Published:** 2024-02-15

**Authors:** D. Líška, S. Rutkowski, L. Oplatková, J. Sýkora, M. Pupiš, J. Novák, E. Urbářová, A. Rutkowska, A. Busch, Alena Kobesova

**Affiliations:** 1 Faculty of Sports Science and Health , Matej Bel University, Banska Bystrica, Slovakia; 2 Faculty of Physical Education and Physiotherapy, Opole University of Technology, Opole, Poland; 3 Department of Rehabilitation Sports Medicine, Second Faculty of Medicine and University Hospital Motol, Charles University, Prague, Czech Republic; 4 Rehabilitation Prague School, Prague, Czech Republic; 5 Department of Health and Human Kinetics, Ohio Wesleyan University, Delaware, OH USA

**Keywords:** Pandemic COVID-19, Physical activity, International physical activity questionnaire (IPAQ)

## Abstract

**Background:**

The COVID-19 pandemic was associated with limited physical activity (PA) of most of the world’s population. This cross-sectional prospective study aimed to assess the levels of PA of university students in Poland, Czech Republic and Slovakia after COVID-19 using the International Physical Activity Questionnaire Short Form (IPAQ-SF).

**Methods:**

A total of 2635 students completed questionnaires regarding their PA levels using the IPAQ-SF between September and December 2022.

**Results:**

PA measured by metabolic equivalent of task (MET) scores, varied between the three countries: Slovakia median MET-minutes/week score 4459.9; Czech Republic 3838.8 Poland 3567.1. The results of the post hoc analysis revealed there were significant differences in MET-minutes/week values between the Czech Republic and Poland (*p* < 0.035) as well as between the Czech Republic and Slovakia (*p* < 0.037). The analysis of energetic expenditure during walking revealed that students from the Czech Republic and Slovakia had higher median MET-min/weeks values (Czech 2284.1; Slovak 2467.1) compared to their Polish (1536.1) peers (*p* < 0.001). Polish cohort presented with significantly higher body mass index (BMI) (*p* < 0.001) than Czech and Slovak groups (BMI Czech: 22.3; Slovak 22.8; Polish 23.8).

**Conclusions:**

Significant differences in PA levels between the Czech Republic, Poland, and Slovakia university students were identified. Slovakia showed the highest median PA measured as a MET score, and Poland showed the lowest. Compared to available pre-COVID studies it seems the total level of PA in the observed cohorts has not returned to the pre-COVID levels and students remain less active.

## Introduction

The coronavirus pandemic (COVID-19) and related public health measures have significantly impacted various aspects of human lives worldwide [1]. Based on the recommendations issued by the World Health Organization (WHO), many restrictions were implemented to reduce the mobility and gathering of people in attempt to secure social distancing. Schools, universities, restaurants and stores were closed for different periods of time with home isolation and curfews ordered in many countries [2–4]. Even outdoor recreation and sports activities were restricted. Generally, physical activity (PA) in the outdoor environment is considered to be very beneficial. Breathing in fresh air can provide higher oxygen levels and better overall respiratory health, exposure to sunlight helps to produce an adequate amount of vitamin D which plays a crucial role in bone health, immune function, and mental well-being. Outdoor environments provide a diverse range of terrains and challenges; thus, walking, running, hiking, or cycling outdoors can engage different muscle groups and improve balance, coordination, and agility. Outdoor PA often provide opportunities for social interaction and exposure to nature can positively impact feelings of happiness, improve mood, self-esteem, vitality, and reduce stress [5, 6]. The WHO published general recommendations for adult PA to eliminate the negative effects associated with mandatory isolation and limited mobility. The recommendations included 150 min of moderate PA or 75 min of vigorous PA per week [7]. Despite these guidelines, several studies indicated a reduction in PA levels in the world population during the pandemic [8–10]. Physical inactivity can be defined as a reduction in PA that causes a reduction in energy expenditure toward the base levels [11, 12]. The European Commission (The Commission’s Directorate General for Health and Food Safety) together with international organizations such as the WHO continuously encourage European countries to increase PA [13, 14]. For centuries, PA has been considered a critical factor in the prevention of chronic diseases, with studies indicating that PA can prevent cardiovascular diseases, diabetes, obesity, various types of cancer, and mental health problems [15–19]. Numerous studies have reported a decline in mental health in all age groups during the pandemic period, noting significant increases in depression, anxiety, insomnia, and overall stress compared to the pre-pandemic time period [20, 21]. These changes were particularly evident among students, who had been recognized as a mental health risk group even before the COVID period [22–25]. Their social and educational patterns were disrupted by the pandemic due to online distance learning, isolation from peers, absence of academic life, and drastically restricted mobility which lasted several months.

The implementation of education programs to increase PA levels to counteract the effects of the pandemic currently remains crucial because some studies warn that younger people could have adopted inactive physical lifestyles during the pandemic and their PA may not return to regular and sufficient amounts after the pandemic [26, 27]. In attempt to further understand the influence COVID-19 has on current PA levels, this study sought to assess and compare the PA levels of university students in Poland, Slovakia, and the Czech Republic post COVID-19 pandemic, when lockdown restrictions were lifted. It was hypothesized that PA levels have not fully returned to pre-pandemic levels.

## Materials and methods

### Participants

This is a cross-sectional prospective study collected via an anonymous online survey. In total, 2635 students participated in the study: 393 from one Polish university, 459 from one Czech Republic university, and 1783 from three Slovakian universities. The study was carried out between September 2022 and December 2022, and information about the study was randomly distributed through social media and electronic learning platforms, with links to a Google Form questionnaire. Only undergraduate and graduate students aged 17 to 30 years, who were native to the relative countries were eligible to participate, while those with visual disabilities and physical disabilities were excluded. All procedures in this study involving human participants were in accordance with the ethical standards of the institutional and national research committee and with the Declaration of Helsinki [28] and its subsequent amendments. Informed consent was obtained from all individual participants involved in the study. The study was approved by the Ethics Committee of Matej Bel University under the number FF 1521/2022.

### International physical activity questionnaire short form (IPAQ-SF)

A validated version of the standardized International Physical Activity Questionnaire Short Form (IPAQ-SF) was used to evaluate the level of PA in the observed sets of university students. It consists of seven questions which inquire about the frequency, duration, and intensity of PA in the last seven days, including walking, moderate-intensity, and vigorous-intensity activities. The IPAQ-SF is a reliable and valid tool to measure PA levels in various populations [29–31], as the IPAQ-SF has been shown to have good test-retest reliability and concurrent validity with other measures of PA, including the Global Physical Activity Questionnaire (GPAQ) [32]. The scoring algorithm for IPAQ-SF includes the calculation of metabolic equivalent of task (MET) minutes per week, which is a standardized unit used to express the energy expenditure of PA.

### Statistical analysis

The Statistica 13 software (StatSoft, Cracow, Poland) and JASP software (JASP Team, Amsterdam, Netherlands) were employed for data analyses. Normality of the data distribution was assessed using the Shapiro-Wilk test, and the significance level was established *a priori* at *p* = 0.05. Not all variables for each country and activity (vigorous activity, moderate activity and walking) level scores demonstrated normal distributions according to Shapiro- Wilk tests, therefore the differences between countries were assessed with a nonparametric One-Way ANOVA (Kruskal-Wallis test). Chi-square tests of independence were also conducted between BMI categories and sex across all three countries.

## Results

Table 1 shows distribution of the studied cohort according to sex, age, body mass index (BMI), height, and weight in the three European countries: Czech Republic (CZ), Poland (PL) and Slovakia (SK). In the overall cohort, a higher proportion of women was observed, but upon examining the distributions within individual countries, it was observed that male groups outnumbered females in Poland and Slovakia. Analysis of BMI, height, and weight revealed significant differences between populations as well. The mean BMI was the lowest in CZ and the highest in PL, suggesting that the Polish population may have a higher prevalence of overweight compared to the other two countries. This was also confirmed after analyzing the association of BMI and sex across all three countries, in which PL demonstrated the greatest percentages of overweight and obese participants for both men and women (Table 2).

**Table 1:** Groups characteristics, *n* = 2635

Variable	Country	Sex	Mean (percentage)	Friedman test
Responses	CZ	Female	321 (69.93%)	
		Male	138 (30.07%)	
		All	459	
	PL	Female	182 (46.31%)	<0.001
		Male	211 (53.69%)	CZ– PL
		All	393	CZ– SK
	SK	Female	826 (46.33%)	
		Male	957 (53.67%)	
		All	1783	
**Variable**	**Country**	**Sex**	**Mean (Standard Deviation)**	**Friedman test**
Age	CZ	Female	22.5 (±3.8)	
		Male	22.4 (±4.0)	
		All	22.5 (±3.9)	
	PL	Female	21.7 (±2.9)	<0.001
		Male	22.5 (±5.0)	SK– PL
		All	22.1 (±4.2)	SK– CZ
	SK	Female	21.5 (±5.0)	
		Male	20.6 (±4.2)	
		All	21.0 (±4.6)	
Height (cm)	CZ	Female	168.5 (±6.1)	
		Male	182.9 (±6.2)	
		All	172.8 (±13.0)	< 0.001
	PL	Female	167.5 (±6.3)	CZ– PL
		Male	180.1 (±6.7)	CZ– SK
		All	174.3 9 (±15.3)	PL– SK
	SK	Female	167.6 (±6.3)	
		Male	180.7 (±7.0)	
		All	174.6 (±14.1)	
Weight (kg)	CZ	Female	61.6 (±9.0)	
		Male	79.4 (±12.2)	
		All	66.9 (±13)	
	PL	Female	63.9 (±12.3)	<0.001
		Male	80.2 (±13.5)	CZ– SK
		All	72.7 (±15.3)	
	SK	Female	62.3 (±11.1)	
		Male	76.5 (±13.2)	
		All	69.9 (±14.1)	
BMI	CZ	Female	21.7 (±2.9)	
		Male	23.7 (±3.3)	< 0.001
		All	22.3 (±3.2)	CZ– PL
	PL	Female	22.8 (±4.1)	PL– SK
		Male	24.7 (±4.1)	<0.018
		All	23.8 (±4.2)	CZ– SK
	SK	Female	22.1 (±3.6)	
		Male	23.4 (±3.7)	
		All	22.8 (±3.7)	

BMI: body mass index; CZ: Czech Republic; PL: Poland; SK: Slovakia

**Table 2:** Associations between sex and BMI categories (observed frequency, [% of participants from given country])

Country	Total (n, %)	BMI Category			
		Underweight (n, %)	Normal (n, %)	Overweight (n, %)	Obese (n, %)
**Czech Republic**	**(459, 17.4)**	**(29, 1.1)**	**(364, 13.8)**	**(54, 2.0)**	**(12, 0.5)**
Female	(321, 69.9)	(28, 6.1)	(262, 57.1)	(27, 5.9)	(4, 0.9)
Male	(138, 30.1)	(1, 0.2)	(102, 22.2)	(27, 5.9)	(8, 1.7)
	*x* ^*2*^ */ p value*		*x* ^*2*^ *= 28.35, p < 0.001, Cramer’s V = 0.249*		
**Poland**	**(393, 14.9)**	**(17, 0.6)**	**(245, 9.3)**	**(94, 3.6)**	**(37, 1.4)**
Female	(182, 46.3)	(15, 3.8)	(125, 31.8)	(30, 7.6)	(12, 3.1)
Male	(211, 53.7)	(2, 0.5)	(120, 30.5)	(64, 16.3)	(25, 6.4)
	*x* ^*2*^ * / p value*		*x* ^*2*^ * = 24.90, p < 0.001, Cramer’s V = 0.252*		
**Slovakia**	**(1783, 67.7)**	**(160, 6.1)**	**(1209, 45.9)**	**(321, 12.2)**	**(93, 3.5)**
Female	(826, 46.3)	(98, 5.5)	(572, 32.1)	(119, 6.7)	(37, 2.1)
Male	(957, 53.7)	(62, 3.5)	(637, 35.7)	(202, 11.3)	(56, 3.1)
	*x* ^*2*^ */ p value*		*x* ^*2*^ *= 27.46, p < 0.001, Cramer’s V = 0.124*		

*Note*: BMI: Body Mass Index

Statistical significance determined *a priori (p* < 0.05)

x^2^ = Chi-square test of independence (r x c) within each country

Strength of association = calculated Cramer’s V

**Fig. 1:**
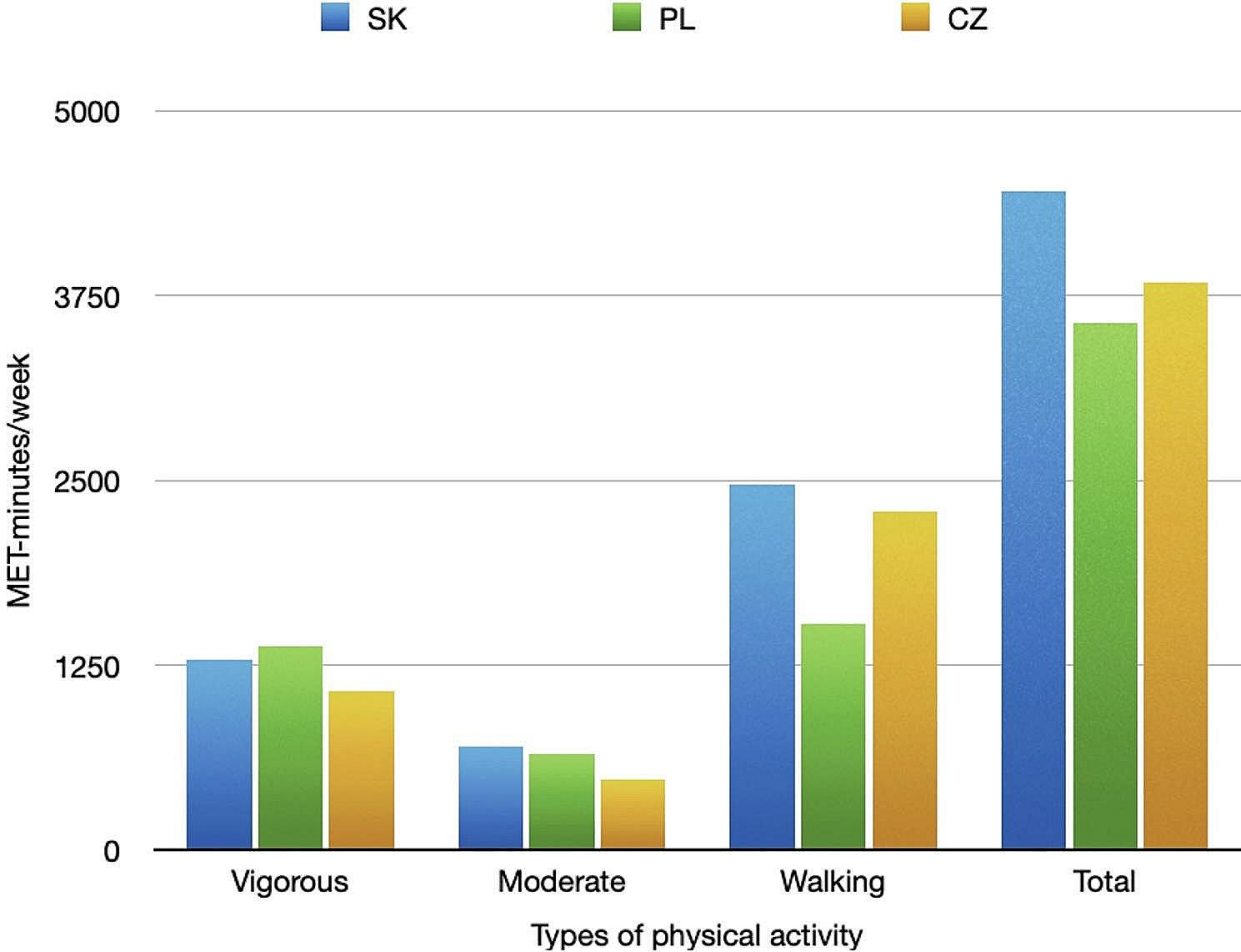
Comparison of physical activity results between countries

PA levels, measured by MET scores, varied between the three countries, with Slovakia showing the highest median MET score and Poland showing the lowest (Table 3). The results of the post hoc analysis revealed there were statistically significant differences in MET values between the Czech Republic and Poland (*p* < 0.035) with (higher MET scores for Czechs) as well as between the Czech Republic and Slovakia (*p* < 0.037) (higher MET scores for Slovakia). When analyzing the levels of vigorous PA by gender across different countries, no statistically significant differences were observed among men. In contrast, among women, statistically significant differences were found between the Czech Republic and Poland (*p* < 0.038) with Czechs showing higher levels of vigorous PA, as well as between the Czech Republic and Slovakia (*p* < 0.002) with Slovakians showing higher levels. In particular, Slovak women demonstrated the highest level of PA compared to women from Poland and Czech Republic. The Slovaks spent significantly less time sitting compared to Poland and the Czech Republic (*p* < 0.001). Figure 1 shows a distribution for the number of minutes of PA between countries.

**Table 3:** The level of physical activity between groups, *n* = 2635

Physical activity	Group, countries	Gender	MET-minutes/week Mean (SD)	Friedman test
Vigorous	CZ	Female	962.1 (±1144.7)	0.135
		Male	1349.0 (±1160.3)	
		All	1078.4 (±1161.8)	
	PL	Female	955.4 (±1422.3)	
		Male	1740.5 (±2023.3)	
		All	1376.9 (±1811.3)	
	SK	Female	1017.8 (±1613.9)	
		Male	1526.8 (±1954.4)	
		All	1291.0 (±1823)	
Moderate	CZ	Female	466.5 (±624.0)	0.728
		Male	498.8 (±697.6)	
		All	476.2 (±646.4)	
	PL	Female	645.7 (±1028.8)	
		Male	661.3 (±1053.3)	
		All	654.1 (±1040.7)	
	SK	Female	570.7 (±1111.8)	
		Male	814.1 (±1369.6)	
		All	701.3 (±1262.3)	
Walking	CZ	Female	2401.6 (±2247.6)	< 0.001
		Male	2010.8 (±1571.3)	
		All	2284.1 (±2073.7)	
	PL	Female	1733.5 (±1383.5)	
		Male	1365.8 (±1226.7)	
		All	1536.1 (±1312.8)	
	SK	Female	2631.9 (±2379.9)	
		Male	2325.0 (±2379.9)	
		All	2467.1 (±2237.9)	
Total	CZ	Female	3830.2 (±2735.5)	<0.001
		Male	3858.7 (±2513.6)	
		All	3838.8 (±2668)	
	PL	Female	3334.6 (±2359.6)	
		Male	3767.6 (±2469.2)	
		All	3567.1 (±2425.6)	
	SK	Female	4220.5 (±3464.0)	
		Male	4665.9 (±3695.9)	
		All	4459.5 (±3596.2)	
Sitting time (% of day)	CZ	Female	0.37 (±0.21)	<0.001
		Male	0.42 (±0.24)	
		All	0.38 (±0.24)	
	PL	Female	0.39 (±0.23)	
		Male	0.43 (±0.25)	
		All	0.41 (±0.24)	
	SK	Female	0.29 (±0.19)	
		Male	0.31 (±0.22)	
		All	0.30 (±0.21)	

The analysis of energetic expenditure during walking revealed that students from the Czech Republic and Slovakia had higher median values compared to their Polish counterparts (2284.1 and 2467.1 vs. 1536.1; *p* < 0.001). These differences were also observed when the data were stratified by gender and compared across the countries, with both men and women from Poland exhibiting significantly lower MET values in the walking domain. There were no significant differences in the values for the mixed population, as well as for each gender, between Czechs and Slovaks.

## Discussion

In this study, significant differences in total PA were identified between the Czech and Polish students, with the Czech university student population showing greater levels of PA, and between the Czech and Slovakian students, with the Slovaks showing greater levels of PA. While male Czech, Slovak, and Polish students appear to be similarly active, Slovak female students are significantly more active than their Czech and Polish peers. The differences in vigorous and moderate PA measured by MET scores between the three countries (Czech Republic, Poland and Slovakia) were not significant. In terms of walking, which is one of the most popular forms of exercise worldwide [33], students from the Czech Republic and Slovakia presented higher median values compared to their Polish counterparts. These differences were also observed when the data were stratified by sex, with both men and women from Poland exhibiting significantly lower MET values in the walking domain. Finally, the total PA levels were highest in males from Slovakia and lowest in females from Poland. Overall, the results suggest there are significant differences in PA levels between the three countries, particularly among women.

Since the Polish education system at secondary schools offers more physical education hours with higher levels of moderate to vigorous adolescent PA during school time as compared with the Czech system [34], one would expect Poles to have a greater adherence to sport and PA, even at university ages. In this context we can also mention the study of Stverakova et al. [35] which identified a significant reduction of PA in Czech school children during COVID. In school settings with low PE hours under normal circumstances [34] and with the longest lockdown (42 weeks) during the pandemic in Europe [35], lower adherence to PA can be expected in the post-COVID times. However, this was not demonstrated in our study; on the contrary, Poles were the least active of the three cohorts studied while also presenting with the highest BMI.

For health benefits, WHO recommends at least 75 min of vigorous PA such as running or 150 min of moderate PA such as brisk walking per week [7] which correlates to 450–600 MET minutes of total activity. This recommended minimum was exceeded 5 to 7 times by all our respondents, which is not surprising in such a young population. Sufficient PA is critically important for a healthy lifespan. In a systematic review by Kyu et al. [36] higher levels of PA were strongly associated with lower risk of serious diseases such as ischemic stroke, ischemic heart disease, breast and colon cancer, and diabetes but only if total PA is several times higher than the recommended minimum level of 600 MET minutes / week, with most health gains occurring at a total activity level of 3000–4000 MET minutes/week. This criterion was achieved by all three observed cohorts (Czech 3838 MET min/week, Polish 3567 and Slovak 4459).

When comparing our results to a study conducted during COVID on 89 Polish university students, which also used the IPAQ, the average reported total PA during April of 2020 was 8640 MET-min/week, and in May 2020 when restrictions were released (“unfreezing period”) the reported PA was 10,560 MET-min/week [9]. It is surprising that even during the lockdown the reported Polish students’ PA was much higher than now in the post-COVID period when it is only 3567 MET-min/week. Polish students also reported much more exercise during COVID (10,560 MET-min/week) [9] than Czechs and Slovaks in this post-COVID study (Czechs 3838.8, Slovaks 4459.5 - See Table 3). The trend of increased PA during COVID is supported by a study with 213 Spanish university students who spent more time performing PA during COVID despite restrictions, but their sitting time was also excessive [37]. The authors of the study emphasized that the sedentary behavior in their cohort of students was already high before the lockdown and there were no significant differences in sitting minutes during the lockdown data collection point. They also concluded that PA of the student cohort increased during lockdown and speculate that students purposefully practiced vigorous PA to compensate for the prolonged sitting time. This finding contradicts a more recent study comparing a larger cohort of Spanish university students’ (*n* = 10,096 participants) PA before-COVID, during the lockdown, and post-COVID [38]. During lockdown, PA significantly reduced (with a significantly larger percentage of students reporting > 7 h per day spent sitting) and BMI values significantly increased relative to before-COVID, both of which returned to non-significant values post-COVID. The authors of both Spanish studies did not report MET-min/week, so unfortunately we cannot exactly compare our PA findings with either study.

Bertocchi et al. [27] used the IPAQ survey to evaluate PA of Italian university students before COVID, during pandemic lockdown and after the COVID in June 2021. A sharp drop in PA during the lockdown was not followed by adequate increases in PA after the restrictions were removed in Italy, as their cohort’s PA had not returned to the pre-pandemic level. Regarding gender, the findings of Bertocchi et al. are in line with our results, as males were more involved in vigorous activities, while females reported greater moderate activities or walking (as in our study). The authors also noted an increase in time spent sitting during the pandemic compared to the pre-pandemic period, and a reduction of sedentary lifestyle observed toward the end of the lockdown when some restrictions were lifted [27].

In studies reporting on mental health, Ács et al. [39] investigated Hungarian university students during the pandemic period and noted participants self-reported worse mental health compared to physical health. These authors observed a significant decrease in total PA and walking times, with an increase in time spent sitting during home confinement. The study did not find significant differences between sexes when comparing total PA values before and during COVID-19. Higher levels of PA were associated with better physical health, while better mental health was positively correlated with the walking domain and total PA. In our study, Polish students walked significantly less than Czech and Slovak students. Another study by Bergier et al. [40] explored the levels of PA in Visegrad countries (Hungry, Slovakia, Czech Republic, and Poland) and Ukraine, using the IPAQ long questionnaire and analyzed the data by gender. Similar to our study, lower PA levels were observed in women (mean PA levels of 5190 MET min/week) compared to men (6023.9 MET min/week). In the Visegrad countries, the mean total PA was 5588.5 MET min/week (i.e., more than we found in our study for Czech, Polish, and Slovak groups) while in Ukraine it was 4233.4. The authors attributed the lower levels of PA in Ukraine to different socio-economic conditions. On the other hand, Ukrainians presented with healthier body mass index’s (BMI). The study by Bergier et al. [40] takes into account one more aspect, and that is the positive role of the self-assessment of one’s own physical fitness. A significant positive correlation was identified between the assessment of physical fitness and the amount of PA both for the Visegrad and Ukrainian groups. Essentially, the more people monitor their own activity, the more active they become. It should be noted that the study was published in 2018, before the conflict in Ukraine. Niźnikowska et al. [41] also investigated the levels of PA among 1169 female students from the Visegrad countries (Czech Republic, Slovakia, Hungary and Poland) using the long version of the IPAQ in a pre-COVID time in 2015. Her team also confirmed the significant effect of the physical fitness self-assessment on the level of PA. These authors also considered factors such as BMI, field of study, self-assessment of physical fitness, and leisure time. They found that 80.7% of female students surveyed from the four Visegrad countries met the WHO recommendations for health-related levels of PA, in the domain of recreation, sport and leisure time with MET min/week ranging from 4969.9 to 5278.6 which is generally more than we identified in our cohorts. Underweight persons moved more than others. Similarly to our findings, Poles were more frequently overweight (13.5% from the observed study cohort) comparatively (Slovaks 11.6%, Czech 12.7% and Hungarian 12.2%).

Similar trends have been reported in America. A study by Dunton et al. [42] examined changes in PA levels among participants in the USA during the pre-COVID-19 (February 2020) and early-COVID-19 (April-May 2020) periods using self-reported and device-based PA variables. The results showed significant reductions in vigorous and moderate intensity PA, walking, and steps counts during the early-COVID-19 period compared to the pre-COVID-19 period, incorporating IPAQ-SF. Furthermore, a study by De La Rosa et al. [43] investigated the impact of lockdown on PA and psychological well-being in Columbian students. The analysis showed a significant decrease in total PA during lockdown for both male and female students, but the effect varied between age categories and PA levels. Their results suggest that the lockdown had significant impacts on PA and psychological well-being [43]. According to the findings of a study by Ammar et al. [44], globally there was a decrease in PA during the lockdown period, as evidenced by a reduction of 33.5% in the number of minutes spent on physical exercise per day and a decrease of 34% in the number of minutes spent walking per day. Additionally, there was an increase in sedentary behavior as the number of hours spent sitting per day increased by 33.5%.

The findings from this study support the endorsement of public health recommendations for university students to strive to achieve greater amounts of daily PA. Given the negative health implications that chronic inactivity presents overall, educational programs and/or initiatives should be necessary components of messaging targeting university students in Poland, Slovakia, and the Czech Republic. This study also has several limitations. First, there was a disproportionate representation of respondents across the countries, with 393 from Poland, 459 from the Czech Republic, and 1783 from Slovakia. Therefore in the largest country we collected the fewest questionnaires, and in the smallest country the most, which may bias the results. This difference is likely due to the fact that Slovak researchers distributed the questionnaires to students from three universities, compared to only one Polish and one Czech university which distributed the questionnaires. This likely reached a broader segment of students, and given the higher median PA reported in Slovakian students, there may have been greater interest in the topic, although due to the random distribution of the study questionnaires, we were unable to track the total number of students who did not agree to participate. Second, the Czech cohort consisted of 70% female respondents and 30% of males, whereas sex distribution in Polish and Slovak groups were more equivalent. Third, the Slovak group was younger (mean age 21.0 years) than the Czech (22.5 years) and Polish groups (22.1 years). Finally, the disciplines studied were not taken into account, as a study by Niźnikowska et al. [41] indicated that students studying technical disciplines are less prone to PA than those studying human and medical disciplines. Thus, our research may not be generally applicable for the entire university student populations. Further research is warranted to assess the long-term trends in PA limitations following the COVID-19 pandemic.

## Conclusion

This study found significant differences in PA levels between the three countries (Czech Republic, Poland, and Slovakia), with Slovakia showing the highest median MET score and Poland the lowest. Czech presented with higher MET scores than Poles but lower than Slovaks. These differences were identified for both the mixed cohort as well as for the female subgroups. No significant differences in vigorous PA levels were observed among men in the observed countries. In addition, moderate activities were relatively similar across the regions, but Poles walked much less than Czechs and Slovaks. Polish students had the highest BMI. In all three countries, the general levels of current PA meet the criteria defined by WHO as necessary for health benefits. However, compared to other available studies it seems that post-COVID student populations in the observed three countries have not returned to the pre-COVID PA levels and remain less active. It is important to continue monitoring the levels of PA and implementing strategies to promote PA, particularly among women, in these regions.

## Data Availability

The datasets used and/or analysed during the current study are available from the corresponding author upon reasonable request.

## Abbreviations

BMI: Body Mass Index
CZ: Czech Republic
IPAQ-SF: International Physical Activity Questionnaire Short Form
MET: Metabolic Equivalent of Task
PA: Physical Activity
PL: Poland
SK: Slovakia
WHO: World Health Organization

## References

[1] Park AH, Zhong S, Yang H, Jeong J, Lee C. Impact of COVID-19 on physical activity: a rapid review. J Glob Health. 12:05003. 10.7189/jogh.12.05003 PMC8979477 35493780

[2] Líška D, Barcalová M, Liptáková E, Jančoková Ľ, Vojtaško Ľ, Gurín D (2021). The level of physical activity of university students in Slovakia during COVID– 19 pandemic. Pedagogy Phys Cult Sports.

[3] Liska D, Andreansky M (2021). Rehabilitation and physical activity for COVID-19 patients in the post infection period. Bratislava Med J.

[4] Palacios Cruz M, Santos E, Velázquez Cervantes MA, León Juárez M (2021). COVID-19, a worldwide public health emergency. Rev Clin Esp (Barc).

[5] Dwyer MJ, Pasini M, De Dominicis S, Righi E (2020). Physical activity: benefits and challenges during the COVID-19 pandemic. Scand J Med Sci Sports.

[6] Nguyen J, Brymer E. Nature-based guided imagery as an intervention for state anxiety. Front Psychol. 2018;9. 10.3389/fpsyg.2018.01858 PMC6176042 30333777

[7] Physical activity. https://www.who.int/news-room/fact-sheets/detail/physical-activity. Accessed 1 Jan 2024.

[8] Rubio-Tomás T, Skouroliakou M, Ntountaniotis D (2022). Lockdown due to COVID-19 and its consequences on Diet, Physical Activity, Lifestyle, and other aspects of Daily Life Worldwide: a narrative review. Int J Environ Res Public Health.

[9] Rutkowska A, Kacperak K, Rutkowski S, Cacciante L, Kiper P, Szczegielniak J (2021). The impact of isolation due to COVID-19 on physical activity levels in adult students. Sustainability.

[10] Chtourou H, Trabelsi K, H’mida C, Boukhris O, Glenn JM, Brach M (2020). Staying physically active during the Quarantine and self-isolation period for Controlling and Mitigating the COVID-19 pandemic: a systematic overview of the literature. Front Psychol.

[11] Booth FW, Roberts CK, Laye MJ (2012). Lack of exercise is a major cause of chronic diseases. Compr Physiol.

[12] Booth FW, Roberts CK, Thyfault JP, Ruegsegger GN, Toedebusch RG (2017). Role of inactivity in chronic diseases: evolutionary insight and pathophysiological mechanisms. Physiol Rev.

[13] Sjöström M, Oja P, Hagströmer M, Smith BJ, Bauman A (2006). Health-enhancing physical activity across European Union countries: the Eurobarometer study. J Public Health.

[14] World Health Organisation. Global action plan on physical activity 2018–2030: more active people for a healthier world. 2018.

[15] Adami PE, Negro A, Lala N, Martelletti P (2010). The role of physical activity in the prevention and treatment of chronic diseases. Clin Ter.

[16] Aubert S, Brazo-Sayavera J, González SA, Janssen I, Manyanga T, Oyeyemi AL (2021). Global prevalence of physical activity for children and adolescents; inconsistencies, research gaps, and recommendations: a narrative review. Int J Behav Nutr Phys Activity.

[17] Reiner M, Niermann C, Jekauc D, Woll A (2013). Long-term health benefits of physical activity– a systematic review of longitudinal studies. BMC Public Health.

[18] Bukova A, Hagovska M, Drackova D, Horbacz A, Wasik J, Krucanica L. Awareness of patients suffering from selected chronic diseases of the importance of physical activity in treating their disorders. Phys Activity Rev. 2019;:234–9.

[19] Bukova A, Hagovska M, Horbacz A, Kručanica L, Kuchelová Z (2019). Are patients with selected chronic diseases informed about the benefits of physical activity?. Trends Sport Sci.

[20] Ahorsu DK, Lin C-Y, Imani V, Saffari M, Griffiths MD, Pakpour AH (2022). The fear of COVID-19 scale: development and initial validation. Int J Ment Health Addict.

[21] Chaturvedi SK (2020). Covid-19, Coronavirus and Mental Health Rehabilitation at Times of Crisis. J Psychosoc Rehabil Ment Health.

[22] Ibrahim AK, Kelly SJ, Adams CE, Glazebrook C (2013). A systematic review of studies of depression prevalence in university students. J Psychiatr Res.

[23] Pedrelli P, Nyer M, Yeung A, Zulauf C, Wilens T (2015). College students: Mental Health problems and treatment considerations. Acad Psychiatry.

[24] Rogowska AM, Kuśnierz C, Bokszczanin A, Examining Anxiety L, Satisfaction (2020). General Health, stress and coping styles during COVID-19 pandemic in Polish sample of University students. Psychol Res Behav Manag.

[25] Rutkowska A, Liska D, Cieślik B, Wrzeciono A, Broďáni J, Barcalová M (2021). Stress levels and Mental Well-Being among Slovak students during e-Learning in the COVID-19 pandemic. Healthc (Basel).

[26] McCarthy H, Potts HWW, Fisher A (2021). Physical activity Behavior before, during, and after COVID-19 restrictions: longitudinal smartphone-tracking study of adults in the United Kingdom. J Med Internet Res.

[27] Bertocchi L, Vecchio R, Sorbello S, Correale L, Gentile L, Buzzachera C (2021). Impact of the COVID-19 pandemic on physical activity among university students in Pavia, Northern Italy. Acta Biomed.

[28] World Medical Association (2013). World Medical Association Declaration of Helsinki: ethical principles for Medical Research Involving human subjects. JAMA.

[29] Biernat E. Międzynarodowy Kwestionariusz Aktywności Fizycznej (IPAQ)– wersja polska.

[30] Lee PH, Macfarlane DJ, Lam TH, Stewart SM (2011). Validity of the International Physical Activity Questionnaire Short Form (IPAQ-SF): a systematic review. Int J Behav Nutr Phys Act.

[31] Meh K, Jurak G, Sorić M, Rocha P, Sember V (2021). Validity and reliability of IPAQ-SF and GPAQ for assessing sedentary behaviour in adults in the European Union: a systematic review and Meta-analysis. Int J Environ Res Public Health.

[32] Bull FC, Maslin TS, Armstrong T (2009). Global physical activity questionnaire (GPAQ): nine country reliability and validity study. J Phys Act Health.

[33] Grigoletto A, Mauro M, Maietta Latessa P, Iannuzzi V, Gori D, Campa F (2021). Impact of different types of physical activity in Green Urban Space on Adult Health and behaviors: a systematic review. EJIHPE.

[34] Groffik D, Mitáš J, Jakubec L, Svozil Z, Frömel K (2020). Adolescents’ physical activity in Education systems varying in the Number of Weekly Physical Education Lessons. Res Q Exerc Sport.

[35] Štveráková T, Jačisko J, Busch A, Šafářová M, Kolář P, Kobesová A (2021). The impact of COVID-19 on physical activity of Czech children. PLoS ONE.

[36] Kyu HH, Bachman VF, Alexander LT, Mumford JE, Afshin A, Estep K (2016). Physical activity and risk of breast cancer, colon cancer, diabetes, ischemic heart disease, and ischemic stroke events: systematic review and dose-response meta-analysis for the global burden of Disease Study 2013. BMJ.

[37] Romero-Blanco C, Rodríguez-Almagro J, Onieva-Zafra MD, Parra-Fernández ML, Prado-Laguna MDC, Hernández-Martínez A (2020). Physical activity and sedentary lifestyle in University students: changes during confinement due to the COVID-19 pandemic. Int J Environ Res Public Health.

[38] Hernández-Segura N, Botella-Juan L, Amezcua-Prieto C, Morales-Suárez-Varela M, Mateos-Campos R, Fernández-Villa T (2023). Excess weight in relation to Lifestyle habits in Spanish First-Year University students: differences between pre- and Post-COVID-19—A serial cross-sectional study based on uniHcos Project. Healthcare.

[39] Ács P, Prémusz V, Morvay-Sey K, Pálvölgyi Á, Trpkovici M, Elbert G (2020). EFFECTS OF COVID-19 ON PHYSICAL ACTIVITY BEHAVIOR AMONG UNIVERSITY STUDENTS: RESULTS OF A HUNGARIAN ONLINE SURVEY. Health Probl Civiliz.

[40] Bergier J, Tsos A, Popovych D, Bergier B, Niźnikowska E, Ács P (2018). Level of and factors determining physical activity in students in Ukraine and the Visegrad Countries. Int J Environ Res Public Health.

[41] Niźnikowska E, Bergier J, Bergier B, Ăcs P, Junger J, Salonna F (2019). Factors influencing levels of physical activity among female students from the Visegrad countries. Health Probl Civiliz.

[42] Dunton GF, Wang SD, Do B, Courtney J (2020). Early effects of the COVID-19 pandemic on physical activity locations and behaviors in adults living in the United States. Prev Med Rep.

[43] De la Rosa A, Monterrosa Quintero A, Camacho-Villa MA, Arc-Chagnaud C, de Andrade AGP, Reyes-Correa S (2022). Physical activity levels and Psychological Well-Being during COVID-19 lockdown among University students and employees. Int J Environ Res Public Health.

[44] Ammar A, Brach M, Trabelsi K, Chtourou H, Boukhris O, Masmoudi L et al. Effects of COVID-19 Home Confinement on eating Behaviour and physical activity: results of the ECLB-COVID19 International Online Survey. Nutrients. 2020;12. 10.3390/nu12061583 PMC7352706 32481594

